# Effects of Accelerated Aging on the Performance of Low-Cost Ultrasonic Sensors Used for Public Lighting and Mobility Management in Smart Cities

**DOI:** 10.3390/s22041560

**Published:** 2022-02-17

**Authors:** Antonino Quattrocchi, Damiano Alizzio, Francesco Martella, Valeria Lukaj, Massimo Villari, Roberto Montanini

**Affiliations:** 1 Department of Engineering, University of Messina, C.da di Dio, 98166 Messina, Italy; damiano.alizzio@unime.it (D.A.); francesco.martella@unime.it (F.M.); valeria.lukaj@unime.it (V.L.); roberto.montanini@unime.it (R.M.); 2 Department of MIFT, University of Messina, Viale Ferdinando Stagno d’Alcontres 31, 98166 Messina, Italy; massimo.villari@unime.it

**Keywords:** accelerated aging, Smart Cities, low-cost device, ultrasonic distance sensor, metrological performance, reliability performance, Edge/Cloud environment, measurement uncertainty

## Abstract

In the field of Smart Cities, especially for Smart Street Lighting and Smart Mobility, the use of low-cost devices is considered an advantageous solution due to their easy availability, cost reduction and, consequently, technological and methodological development. However, this type of transducers shows many critical issues, e.g., in metrological and reliability terms, which can significantly compromise their functionality and safety. Such issue has a large relevance when temperature and humidity are cause of a rapid aging of sensors. The aim of this work is to evaluate the effects of accelerated aging in extreme climatic conditions on the performance of a control system, based on a low-cost ultrasonic distance sensor, for public-lighting management in Smart Cities. The presented architecture allows for the detection of vehicles, pedestrians and small animals and contains a dedicated algorithm, developed in an Edge/Cloud environment, that is able to display the acquired measurements to users connected on the web. The obtained results highlight that the effect of accelerated aging is to significantly reduce the linearity of the calibration curve of the sensor and, moreover, to exponentially increase the number of outliers and invalid measurements. These limitations can be overcome by developing an appropriate self-calibration strategy.

## 1. Introduction

The continuous development, at the technological level, of modern society has now led to the exponential spread of smart devices and infrastructures in every aspect of common life and recently also in urban environments [1,2,3,4]. The European Commission defines Smart Cities as cities using technological solutions to improve the management and efficiency of the urban environment [5]. These are modern cities in which a network of sensors and actuators, integrated throughout the urban area, is able to interact with wireless mobile devices [6]. In this way, it is possible to exchange a large amount of data, useful for optimizing several services such as controlling road traffic, recording weather and pollution conditions and monitoring the structural health of buildings and infrastructures [7,8,9,10,11].

Recently, in the context of urban construction and road expansion, various aspects have emerged. Mainly, they focus on energy management and saving [12,13]. Indeed, it is estimated that public street lighting currently requires about 35 TWh of electricity, with over 56 million of streetlamps only for the international urban environment [14]. This involves the introduction of further adaptations relating to cost reduction, increased efficiency and digitization improvement, currently identified by the term Smart Street Lighting [15] in Smart Mobility background [16].

The use of low-cost sensors and actuators is an extremely advantageous solution in terms of easy availability and cost reduction [17,18]. However, in the field of Smart Cities, low-cost devices show several critical issues, including limited operative reliability and a large uncertainty of the acquired and transmitted measurement [19,20,21]. To overcome these limitations, several solutions have been proposed in the literature, for example, redundancy installation or on-site recalibration [22,23]. Glass et al. [24] evaluated the applicability of low-cost sensors for the analysis of atmospheric pollution, developing and analysing a device consisting of two CO and NO_2_ sensors and a further two certified reference sensors. The comparison between the performed measurements highlighted a determination coefficient of lower than 0.72, which increased to 0.83 after an appropriate recalibration of the low-cost sensors. Idrees et al. [25] implemented algorithms to avoid faults due to the temporary malfunction and to manage cross-sensitivity issues in low-cost sensors for detecting air quality. Although several advances have been highlighted, both the cited papers reported an increase in terms of necessary economic resources and data to be processed.

In addition, the metrological performance of low-cost sensors is also affected by aging, for example, by atmospheric agents. Although this is a topic well addressed by several researchers on different types of devices and measurement systems [26,27], few studies have been published in the case of low-cost sensors. Tryner et al. [28] evaluated the performance of low-cost particulate sensors, exposed to high concentrations of road dust for 18 h. Despite the reduced aging time, some of the analysed devices showed consistent results, while others began reporting erroneously high values. Samad et al. [29] investigated the effect of temperature and humidity on low-cost gas sensors, reaching critical conditions of 85% of RH and 45 °C. In this case, a significant decrease in data quality was highlighted, which was subsequently revised with a designed correction algorithm.

The field of low-cost ultrasonic distance sensors is less oriented towards estimating the accuracy of the measurements. Vakula et al. [30] developed a parking system based on the use of low-cost distance sensors able to identify the car occupancy for an online booking infrastructure. In this case, the measurement performance was not investigated, considering as optimal the datasheet provided by the manufacturer of the low-cost sensor. Jamaluddin et al. [31] implemented a distance measurement system that can be directly connected to a smartphone, only verifying its ability to display the acquired signal. Indeed, the results underline how a high accuracy of the static measurement is achieved without having sufficiently exhaustive tests and, above all, validated the performance of the used sensor. Nuryanto et al. [32] demonstrated the applicability of such a family of sensors for the autonomous driving of a robot, without, however, taking into account the quality of the presented results. A first result in terms of measurement quality was obtained by Zhmud et al. [33]. They compared different ultrasonic sensors and identified the one with the best characteristics. Finally, to the best of our knowledge, we have not found any studies on the effect of aging on low-cost ultrasonic sensors.

In summary, the state-of-the-art clearly manifests the need to use low-cost sensors for large implementations, but, at the same time, it has not fully addressed the problem of measurement and reliability over time of these devices.

The aim of this paper is to investigate the effects of accelerated aging on the metrological performance of a control system, based on a low-cost ultrasonic distance sensor, for public lighting management in the field of Smart Street Lighting and Smart Mobility. Therefore, we engaged an accelerated aging process in extreme climatic conditions (70 °C and 90% of HR) for 21 days to develop algorithms able to periodically and remotely self-calibrate such devices, guaranteeing the best measurement results. Specifically, the analysis has been also performed to evaluate the accuracy of the target distance and the reliability of the employed measurement systems.

## 2. Materials and Methods

### 2.1. Low-Cost Ultrasonic Distance Sensor (HY-SRF05)

**Figure 1:**
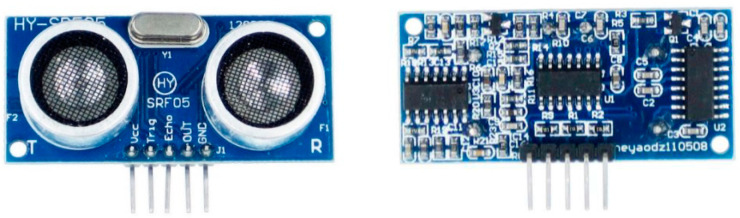
Low-cost ultrasonic distance sensor (HY-SRF05). R and T indicate the receiver and transmitter, respectively.

The investigated device (Figure 1) was a low-cost ultrasonic distance sensor (HY-SRF05), consisting of a transmitter, a receiver and an integrated microprocessor. It was equipped with five pins with the following functions: V_cc_ and *GND* pins were used for its power supply, *Trig* pin receives the start input of the measurement process, *Echo* pin transfers the signal useful for the same measurement to the acquisition system and *OUT* pin, which was not employed in this study, allows a bidirectional input/output wire. All communication pins (*Trig*, *Echo* and *OUT*) use 0–5 V digital signals.

The HY-SRF05 allows for the estimation of the distance between itself and a solid and sufficiently flat target. Hence, it is adapted to detect a vehicle, a pedestrian and some small animals. Its datasheet is reported in Table 1.

As indicated in Table 1, HY-SRF05 is able to detect a target up to the maximum distance of 450 cm. For this reason, it could perform its function if placed on a lighting pole. In such a way, it would have the advantage of being easily supplied and connected by LAN. However, considering the limited measurement range, in some cases, its use would lead to an inappropriate activation of the lighting control system. In fact, due to the speed of a vehicle, only the poles following the one equipped with the sensor could be adequately controlled. This disadvantage could be overcome or limited through the installation of measuring columns or the application of control systems based on specific algorithms. On the other hand, in the case of pedestrians or small animals, no particular issues would appear.

The distance *d* between the HY-SRF05 and the target is calculated by measuring the time-of-flight *t* of the generated ultrasonic wave, i.e., the time required for the ultrasonic wave to reach the target, to be reflected and to return to the receiver. This happens according to Equation (1):

(1) $$d=\frac{v_{s}t}{2}$$

where *v_s_* represents the speed of sound in air at room temperature (25 °C) [34]. Furthermore, the signal produced by the HY-SRF05 does not propagate on the plane, but in space and by tracing a cone. This feature produces, on the one hand, an analysing area that can be adjusted with the distance, and, on the other, the need to place the device at a specific height from the ground. For such reasons, if these sensors are integrated on the lighting poles, they should be placed at suitable heights and with specific inclinations. Therefore, in order to correctly identify the presence of vehicles, pedestrians and small animals, the authors estimate that the minimum height, at which the sensor must be positioned, should be 200 cm. Furthermore, 200 cm could be a sufficient distance to activate a lighting control system following the particular detection of pedestrians and small animals.

**Figure 2:**
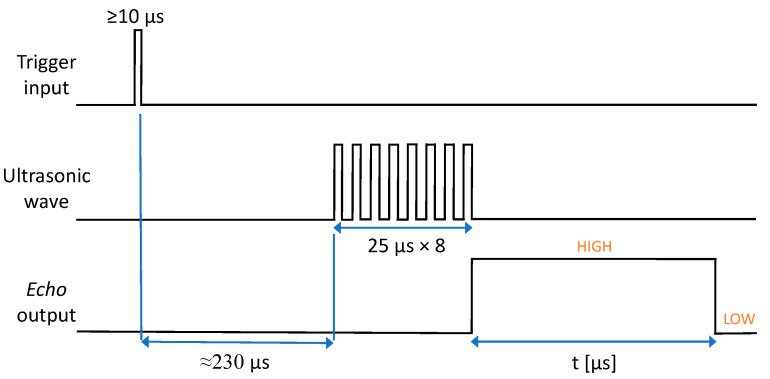
Operation scheme of the HY-SRF05.

The operation scheme of the HY-SRF05 provides that the sensor is powered and managed by an external microcontroller. The measurement procedure (Figure 2) starts when a *HIGH* pulse, lasting at least 10 μs, is sent to *Trig* pin of the sensor. This allows for the integrated microprocessor to active the transmitter in order to generate a train of eight pulses, each of them with a duration of 25 μs, and to set the output of *Echo* pin in the *HIGH* status. Simultaneously, the time count starts and stops only when the ultrasonic wave, reflected by the target, reaches the receiver. Once this is done, the output of *Echo* pin returns to *LOW* status. The duration of *HIGH* period hence represents the time-of-flight *t* of the ultrasonic signal in Equation (1). It should be noted that the generation of the train of eight pulses takes about 230 μs.

### 2.2. Experimental Setup and Method

**Figure 3:**
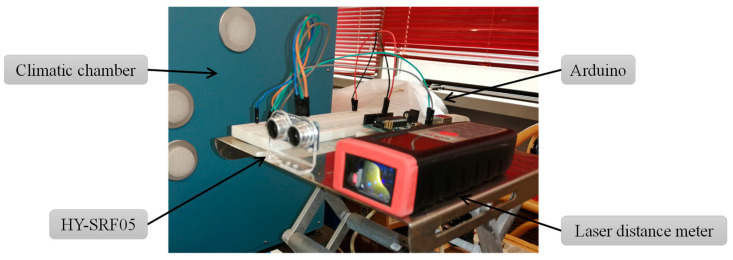
Detail of the experimental setup.

The experimental setup (Figure 3) included a HY-SRF05 and a polypropylene target with dimensions of 500 mm × 500 mm × 5 mm on a mobile tripod placed in front of the same sensor. The target–sensor distance was adjusted by means of a laser distance meter (mod. WDM 100, Würth, Künzelsau, Germany). The acquisition system consisted of an Arduino mod. Uno rev.3, connected to a single-board computer Raspberry mod. Pi 3 B + and that on-line via LAN cable. The acquisition frequency was set at 45 Hz.

**Figure 4:**
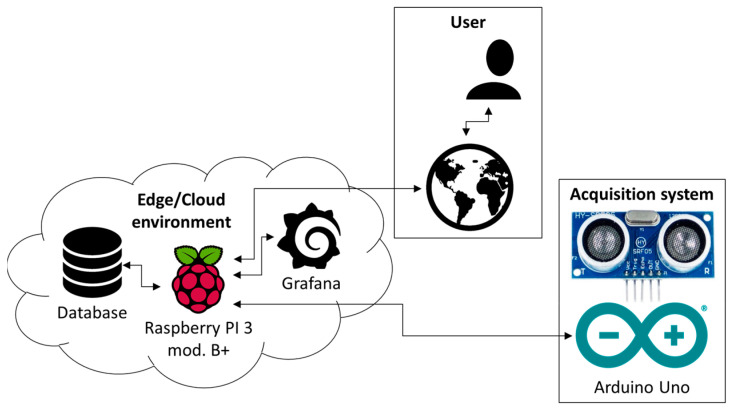
Representative scheme of the acquisition system in an Edge/Cloud environment.

The management procedure of the HY-SRF05, the measurement of the time-of-flight *t* and the computation of the distance *d* were implemented by a sketch on Arduino, while on Raspberry an Edge/Cloud environment, including the software for data communication, an NoSQL database and a software installation by Grafana, was placed [35,36,37]. Specifically, the Grafana platform, accessible from the Web, allows to view distance results, control the acquisition system and send information to the operator/user. Figure 4 schematically describes the adopted method.

The testing phase involved an accelerated aging of the HY-SRF05 by a climatic chamber (mod. 2500, Thunder Scientific) able to guarantee a constant temperature of (70 ± 0.1) °C and a relative humidity of (90 ± 0.1)% HR for 21 days. Periodically, the sensor was removed from the chamber, brought back to ambient conditions, (25 ± 1) °C and (50 ± 5)% of HR, waiting for 15 min, before the measurements of the target distance were carried out. Specifically, the effects of the climatic stress on full scale of the HY-SRF05 was evaluated.

## 3. Results

**Figure 5:**
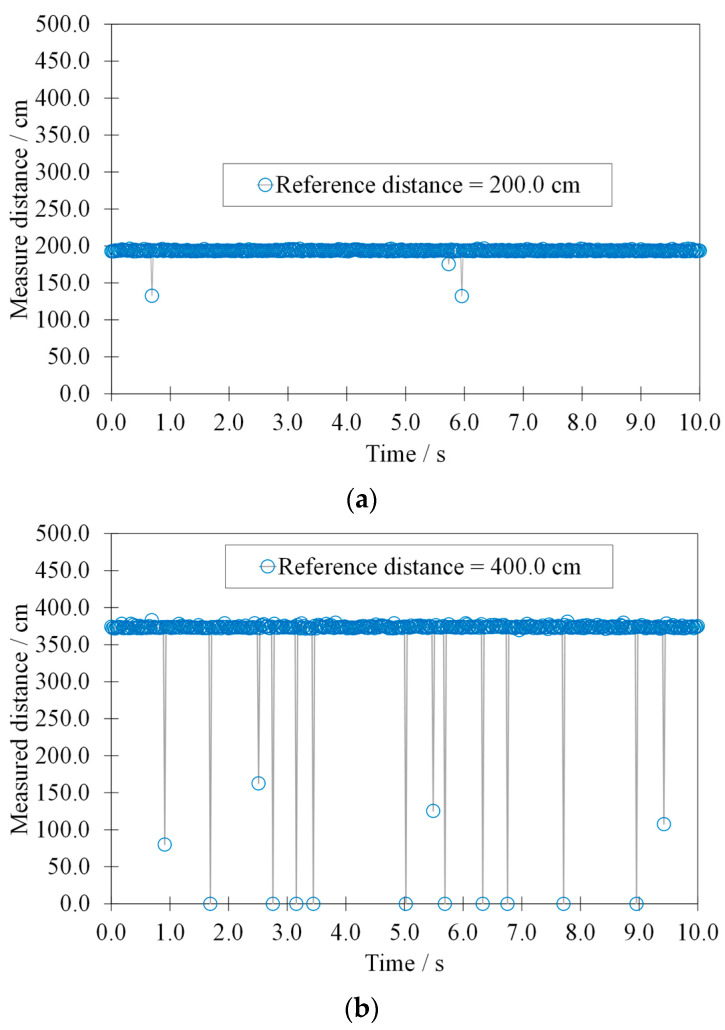
Typical signals: measured distance by the HY-SRF05 at a reference distance of (**a**) 200.0 cm and of (**b**) 400.0 cm as a function of time, before the effects of the accelerated aging.

Figure 5a,b show the typical signals relating to the measured distances by the HY-SRF05, before the latter was subjected to the accelerated aging.

The acquired signals have good stability, which becomes reduced as the target–sensor distance increases. Indeed, some measurements have a value significantly below the average. These evolve rapidly in outliers (amplitude less than 50% of the average of the signal) and, subsequently, also in invalid measurements (amplitude equal to 0 mm). The presence of outliers and invalid measurements could be attributed, respectively, to computational errors of the integrated microprocessor of the HY-SRF05 and to non-reception of the ultrasound wave reflected by the target.

**Figure 6:**
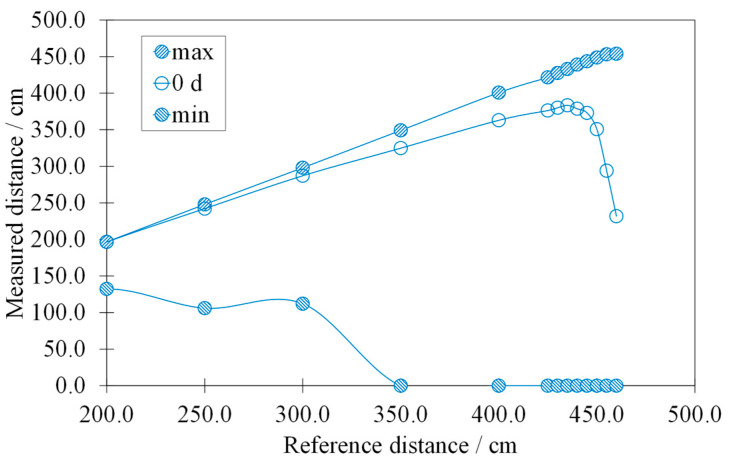
Calibration curve of the HY-SRF05, before the latter is subjected to the accelerated aging (0 d), with the curves of the maximum (max) and minimum (min) values.

Figure 6 illustrates the calibration curve of the HY-SRF05, before the latter was subjected to accelerated aging (0 d).

Each point of the calibration curve corresponds to an average of 450 values, acquired in 10 s at a specific reference distance. Instead, the maximum and minimum curves connect, respectively, the maximum and minimum points calculated at the defined reference distances. The calibration curve maintains a good linearity at up to 435.0 cm, and after that there is a fast reduction. This behavior is related to the presence of outliers and invalid measurements as the target–sensor distance increased. It depends not only on the accuracy of the analyzed sensor, but also on its reliability since increasing reference distances correspond to decreasing measured distances.

**Figure 7:**
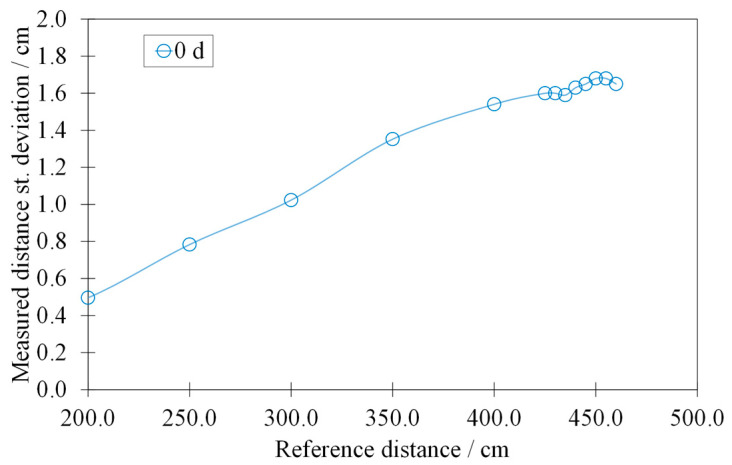
Standard deviation of the calibration curve of the HY-SRF05 without outliers and invalid measurements.

Figure 7 displays the trend of the standard deviation, calculated for the points of the static characteristic, without outliers and invalid measurements.

By eliminating the outliers and the invalid measurements from the acquired signal, we obtained an increasing standard deviation of much greater than the resolution declared by the manufacturer (i.e., 0.3 cm). Furthermore, it would seem that its trend is non-linear with respect to the measured distance.

**Figure 8:**
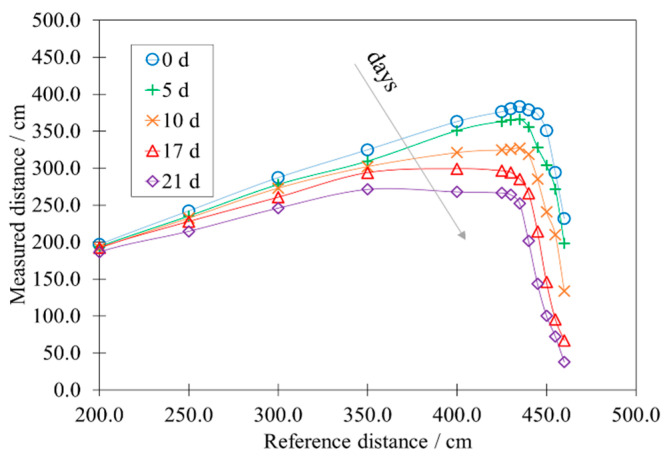
Influence of the accelerated aging on the calibration curve of the HY-SRF05.

Figure 8 reports the effect of accelerated aging on the calibration curve of the HY-SRF05.

As the time of treatment at high temperature and humidity continues, the sensor exhibits a notable decline in its performance. The linearity of the measurement range is dramatically reduced with a large consequent decrease in the average of the measured values. For example, at 21 d of aging, the sensor reaches a full scale of 350 cm. After that, the HY-SRF05 becomes unreliable.

**Figure 9:**
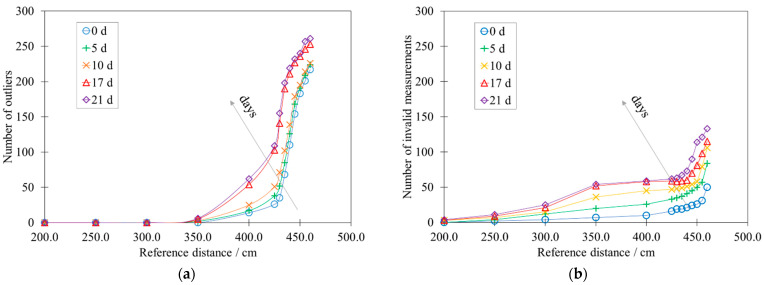
Influence of the accelerated aging on (**a**) the number of outliers and on (**b**) the number of invalid measurements.

Figure 9a,b highlight the influence of the accelerated aging on the number of outliers and invalid measurements, respectively.

The number of outliers (Figure 9a) increases non-linearly, following a first exponential and a second logarithmic segment, while that of the invalid measurements (Figure 9b) seems substantially exponential. It is clear that the effect of the accelerated aging is to increase the slope of these trends.

**Figure 10:**
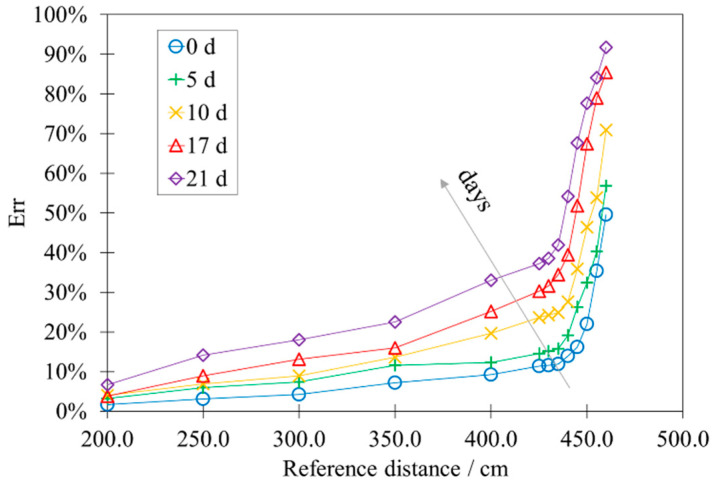
Influence of the accelerated aging on the percentage error *Err*.

Figure 10 considers the trend of the percentage error *Err* vs. the reference distance. *Err* is calculated considering the following Equation (2):

(2) $$Err\;\left(\%\right)=\frac{d_{ref}-\bar{d}}{d_{ref}}$$

where *d_ref_* is the reference distance and $\bar{d}$ is the average measured distance (see Figure 7).

*Err* has a growing exponential trend, for which the speed increases as the accelerated aging period increases. Its increase is correlated to that of the number of outliers and invalid measurement, occurring after the same accelerated aging.

Appendix A exhibits the data relating to Figure 9a,b and Figure 10 in Table A1, Table A2 and Table A3.

## 4. Discussion and Conclusions

The paper explores the performance of a control system, based on a low-cost ultrasonic distance sensor (HY-SRF05), employed for public lighting management in Smart Cities and, consequently, in the detection of vehicles, pedestrians and also small animals.

The study highlighted the presence of some significant limitations in the use of these devices, proposing quantitative data for their metrological properties. First of all, the calibration curve of the HY-SRF05 shows a lower linearity range than the declared one. In the same way, the standard deviation of the points of the calibration curve is greater than the declared resolution and is not constant but presents an increasing trend. In addition, sporadic acquisition errors (outliers and invalid measurements) were identified. The effect of accelerated aging is to reduce the performance of the HY-SRF05, significantly influencing the linearity of the calibration curve and exponentially increasing the number of outliers and invalid measurements. These results lead to a decline in measurement accuracy and reliability, which are significantly relevant in the field of Smart Street Lighting. Indeed, errors in the control of switching on, switching off and light intensity can be responsible not only for inefficiencies but also for a substantial reduction in the safety of people and things.

A promising solution could be the introduction of an appropriate self-calibration strategy, adopting innovative Machine Learning algorithms at the Edge, which are able to reduce these shortcomings and increase the longevity of the proposed measurement system.

## Figures and Tables

**Table 1:** Datasheet of HY-SRF05.

Parameter	Value
Measurement range	2–450 cm
Resolution	0.3 cm
Detection angle	±15°
Supply voltage	5 V in DC
Supply current	2 mA
Output signals	HIGH, 5 V in DC LOW, 0 V in DC
Working temperature	From −20 °C to +60 °C
Dimensions (length × width × thickness)	45 mm × 20 mm × 10 mm
Weight	10 g

## Data Availability

Not applicable.

## Appendix A

Table A1, Table A2 and Table A3 reports the data plotted in Figure 9a,b and Figure 10, respectively.

**Table A1:** Influence of the accelerated aging on the number of outliers (Figure 9a).

Reference Distance [mm]	Number of Outliers				
	at 0 d	at 5 d	at 10 d	at 17 d	at 21 d
200.0	0	0	0	0	0
250.0	0	0	0	0	0
300.0	0	0	0	0	0
350.0	0	2	3	5	6
400.0	14	17	25	54	62
425.0	26	38	51	103	109
430.0	35	52	71	141	155
435.0	68	85	102	190	198
440.0	110	126	139	211	219
445.0	154	168	179	227	232
450.0	183	191	195	236	240

**Table A2:** Influence of the accelerated aging on the number of invalid measurements (Figure 9b).

Reference Distance [mm]	Number of Invalid Measurements				
	at 0 d	at 5 d	at 10 d	at 17 d	at 21 d
200.0	0	0	3	3	4
250.0	2	4	7	9	11
300.0	4	12	15	21	25
350.0	7	20	36	52	54
400.0	10	26	45	58	59
425.0	16	33	47	59	62
430.0	19	35	48	58	63
435.0	19	37	49	59	67
440.0	21	41	51	60	73
445.0	24	45	54	70	90
450.0	26	50	58	81	114

**Table A3:** Influence of the accelerated aging on the percentage error *Err* (Figure 10).

Reference Distance [mm]	*Err*				
	at 0 d	at 5 d	at 10 d	at 17 d	at 21 d
200.0	2%	3%	4%	4%	7%
250.0	3%	6%	7%	9%	14%
300.0	4%	7%	9%	13%	18%
350.0	7%	12%	14%	16%	22%
400.0	9%	12%	20%	25%	33%
425.0	11%	15%	24%	30%	37%
430.0	12%	15%	24%	32%	39%
435.0	12%	16%	25%	34%	42%
440.0	14%	19%	28%	39%	54%
445.0	16%	26%	36%	52%	68%
450.0	22%	32%	46%	68%	78%
